# Feed-Forward Inhibition of CD73 and Upregulation of Adenosine Deaminase Contribute to the Loss of Adenosine Neuromodulation in Postinflammatory Ileitis

**DOI:** 10.1155/2014/254640

**Published:** 2014-08-19

**Authors:** Cátia Vieira, Maria Teresa Magalhães-Cardoso, Fátima Ferreirinha, Isabel Silva, Ana Sofia Dias, Julie Pelletier, Jean Sévigny, Paulo Correia-de-Sá

**Affiliations:** ^1^Laboratório de Farmacologia e Neurobiologia, UMIB and MedInUP, Instituto de Ciências Biomédicas Abel Salazar (ICBAS), Universidade do Porto (UP), L. Prof. Abel Salazar 2, 4099-033 Porto, Portugal; ^2^Centre de Recherche du CHU de Québec, CHUL, Québec, QC, Canada G1V 4G2; ^3^Département de Microbiologie-Infectiologie et d'Immunologie, Faculté de Médecine, Université Laval, Québec, QC, Canada G1V 0A6

## Abstract

Purinergic signalling is remarkably plastic during gastrointestinal inflammation. Thus, selective drugs targeting the “purinome” may be helpful for inflammatory gastrointestinal diseases. The myenteric neuromuscular transmission of healthy individuals is fine-tuned and controlled by adenosine acting on A_2A_ excitatory receptors. Here, we investigated the neuromodulatory role of adenosine in TNBS-inflamed longitudinal muscle-myenteric plexus of the rat ileum. Seven-day postinflammation ileitis lacks adenosine neuromodulation, which may contribute to acceleration of gastrointestinal transit. The loss of adenosine neuromodulation results from deficient accumulation of the nucleoside at the myenteric synapse despite the fact that the increases in ATP release were observed. Disparity between ATP outflow and adenosine deficit in postinflammatory ileitis is ascribed to feed-forward inhibition of ecto-5′-nucleotidase/CD73 by high extracellular ATP and/or ADP. Redistribution of NTPDase2, but not of NTPDase3, from ganglion cell bodies to myenteric nerve terminals leads to preferential ADP accumulation from released ATP, thus contributing to the prolonged inhibition of muscle-bound ecto-5′-nucleotidase/CD73 and to the delay of adenosine formation at the inflamed neuromuscular synapse. On the other hand, depression of endogenous adenosine accumulation may also occur due to enhancement of adenosine deaminase activity. Both membrane-bound and soluble forms of ecto-5′-nucleotidase/CD73 and adenosine deaminase were detected in the inflamed myenteric plexus. These findings provide novel therapeutic targets for inflammatory gut motility disorders.

## 1. Introduction

The enteric nervous system (ENS) undergoes a series of adaptive responses to different pathological conditions (e.g., inflammatory and/or ischemic insults) [1, 2]. For instance, enteric neurons rapidly change their structure, function, or chemical phenotype in order to maintain gut homeostasis. Even if the inflammatory insult is brief and the damage circumscribed, its repercussion on enteric neurons may be long-lasting leading to significant changes in intestinal function, which can be observed in remote regions from the inflammation site. The postinflammatory status is frequently accompanied by substantial increases in enteric motility [3].

Inflammation of the gastrointestinal (GI) tract causes marked changes in the release of purines leading to subsequent adaptive modifications of purinoceptors expression and/or function (reviewed in [4]). The underlying mechanisms of disturbed purinergic modulation are not completely understood, in part because the study of purinoceptors may be hampered by the presence of distinct nucleotide release sites and cell surface enzymes that rapidly break down extracellular nucleotides into nucleosides [5]. In healthy individuals, ATP is released predominantly from stimulated enteric neurons [6], but its release from nonneuronal cells (e.g., smooth muscle fibres, interstitial cells of Cajal) might also occur [7]. Four members of the ecto-nucleoside triphosphate diphosphohydrolase (E-NTPDase) family, namely, NTPDase1, NTPDase2, NTPDase3, and NTPDase8 and two members of the ecto-nucleotide pyrophosphatases/phosphodiesterases (E-NPP) family, NPP1 and NPP3, are located at the plasma membrane and hydrolyse extracellular nucleotides [5, 8, 9]. The relative contribution of distinct ecto-enzymes to the modulation of purinergic signalling depends not only on differential tissue and cell distribution, regulation of expression, and targeting to specific membrane domains but also on substrate preference and availability.

Regarding the substrate preference, NTPDase1 (CD39 or apyrase) dephosphorylates ATP directly into AMP, with minimal accumulation of ADP. NTPDase2 (ATPase) is a preferential nucleoside triphosphatase that hydrolises ADP 10 to 15 times less efficiently than ATP, leading to minimal AMP accumulation [8]. NTPDase3 and NTPDase8 are functional intermediates between NTPDase1 and NTPDase2 [8]. Because of their involvement in physiological processes, namely, blood clotting, vascular inflammation, immune reactions, and certain types of cancer, NTPDases are now considered as potential drug targets [10]. As for NPP1 and NPP3, they release nucleoside 5′-monophosphate from a variety of nucleotides, but intriguingly, their phosphorylated product (e.g., AMP) binds to NPPs with a higher affinity than substrates do and thus inhibits catalysis [9]. Finally, AMP is hydrolysed to adenosine and inorganic phosphate by ecto-5′-nucleotidase (CD73), which is concentrated in the myenteric smooth muscle cell layer [7]. Interestingly, ecto-5′-nucleotidase (CD73) may be cleaved from cell membranes through hydrolysis of the GPI anchor by phosphatidylinositol-specific phospholipases or by proteolysis while retaining its catalytic activity in the soluble form [11].

At the myenteric neuromuscular synapse, ATP is primarily metabolized into AMP, which is then dephosphorylated into adenosine; alternative conversion of ATP into ADP is more relevant at high ATP concentrations [12]. Thus, gradients of ATP and its breakdown products (ADP and adenosine) may provide fine tuning control of peristaltic motor performance in the gut during stressful situations, such as sustained neuronal activity, ischemia, and inflammation, when extracellular ATP reaches high levels (see [13, 14]). ATP transiently facilitates acetylcholine (ACh) release from nonstimulated nerve terminals via prejunctional P2X (probably P2X2) receptors. Hydrolysis of ATP directly into AMP and subsequent formation of adenosine activates inhibitory A_1_ receptors in smooth muscle fibers [15] and in ganglionic cell bodies of myenteric neurons [16], where it downmodulates the release of excitatory neurotransmitters, such as substance P [17] and ACh [18]. Besides the well characterized inhibitory A_1_ receptors, myenteric nerve terminals are endowed with prejunctional A_2A_ receptors mediating facilitation of ACh release [16, 18, 19].

Previous findings from our group demonstrated that the ectonucleotidase pathway contributes only partially to the total interstitial adenosine concentration in the myenteric plexus [19]. Adenosine released as such from either neuronal or nonneuronal cells seems to be the main source of endogenous adenosine in the enteric nervous system [18]. This release is sought to be mediated by facilitated diffusion via nucleoside transporters, which can be regulated by endogenous signaling molecules, such as ACh via muscarinic M_3_ receptors [16, 19]. On the other hand, we demonstrated that the extracellular deamination of adenosine by adenosine deaminase (ADA) represents the most effective mechanism regulating synaptic adenosine levels in the myenteric plexus-longitudinal muscle (LM-MP) of the ileum from healthy animals [18, 19]. ADA is a ubiquitous purine metabolic enzyme localized on the cell surface (ecto-ADA) [20]. Upon proteolytic cleavage from cell membranes, soluble forms of ADA (exo-ADA) retain their catalytic activity and may be found in interstitial fluids [21]. We detected a relatively high ADA activity (~0.6 U/mL) in superfusates collected after stimulating LM-MP preparations, which is in keeping with the hypothesis that “ADA secretion” may restrict endogenous adenosine actions to the synaptic region near the release/production sites [19]. ADA secretion increases in various diseases and may be originated from monocyte/macrophage lineages, thus reflecting the involvement of the cellular immune system. de Man and colleagues reported that chronic intestinal inflammation enhanced the enteric contractile activity in part due to an impairment of purinergic modulation of cholinergic nerve activity [14].

Because, adenine nucleotides and nucleosides (and ADA itself) may be released from activated inflammatory cells [22], as well as from neighbouring neuronal and nonneuronal enteric cells [23, 24], there is an increasing interest in the neuromodulatory effects exerted by adenosine during inflammatory insults. The therapeutic potential of adenosine-related compounds for controlling intestinal motility and inflammation [14, 25] prompted us to investigate the kinetics of adenosine formation/inactivation cycle in order to understand the overall homeostatic role of the nucleoside on enteric excitability in postinflammatory ileitis caused by intraluminal instillation of TNBS in the rat.

## 2. Material and Methods

### 2.1. Animals

Rats of either sex (Wistar, ~200 g) (Charles River, Barcelona, Spain) were kept at a constant temperature (21°) and a regular light- (06.30–19.30 h) dark (19.30–06.30 h) cycle, with food and water) and a regular light- (06.30–19.30 h) dark (19.30–06.30 h) cycle, with food and water* ad libitum*. All studies involving animal experiments are reported in agreement with ARRIVE guidelines. Animal handling and experiments were in accordance with the guidelines prepared by Committee on Care and Use of Laboratory Animal Resources (National Research Council, USA) and followed the European Communities Council Directive (86/609/EEC).

### 2.2. TNBS-Induced Intestinal Inflammation Model

Intestinal inflammation was produced by the instillation of 2,4,6-trinitrobenzenesulfonic acid (TNBS) into the lumen of the rat ileum. After a fasting period of 4–8 hours with free access to drinking water, rats undergo median laparotomy under anaesthesia with an association of medetomidine (10 mg/Kg) and ketamine (75 mg/Kg) given subcutaneously. At the end of the procedure animals were retrieved with atipamezole (10 mg/Kg). The terminal ileal loop was gently exteriorized, and TNBS (40 mM) was injected through the enteric wall into the lumen of the ileum, 10 centimeters proximal to the ileocolonic junction. Controls received 1 mL of 0.9% saline. Sixty minutes after surgery, the rats were allowed to eat and drink* ad libitum*. After surgery, pain was controlled with tramadol hydrochloride (10 mg/Kg). To have a control of time course of body weight loss and recovery after injection of TNBS, rats were weighed prior to TNBS administration and daily following surgery. Animals with intestinal inflammation (TNBS) transiently lose weight for three to four days after surgery and regain weight thereafter. Seven days after surgery, animals were sacrificed following an overnight fasting period.

### 2.3. Histological Findings and Gastrointestinal Motility

The postinflammatory phase of TNBS-induced ileitis was characterized in haematoxylin-eosin stained sections using Pontell and Jergens criteria, which is based in the loss of normal tissue architecture, epithelial damage, and infiltration of inflammatory cells [3, 26–28]. Evaluation of gastrointestinal (GI) motility was performed* in vivo* by oral administration of a gavage containing the methylene blue dye. Dye progression in the GI tract was evaluated after 30 minutes, both in control and TNBS-injected animals: the total length of the small intestine was used to normalize data.

### 2.4. [^3^H]-Acetylcholine Release Experiments

Sections of the rat ileum not including the terminal five centimetres were used. The longitudinal muscle strip with the myenteric plexus attached was separated from the underlying circular muscle according to the method of Paton and Vizi [29]. This preparation is highly enriched in cholinergic neurons, mainly excitatory neurons projecting to the longitudinal muscle (25%) that receive inputs from intrinsic primary afferents (26%) and from ascending and descending pathways (17%) [30].

The procedures used for labelling the preparations and measuring evoked [^3^H]-Acetylcholine ([^3^H]-ACh) release were previously described [12, 16, 18, 19] and used with minor modifications. LM-MP strips were mounted in 3 mL capacity vertical perfusion chambers heated at 37°C. After a 30 min equilibration period, myenteric neurons were labelled for 40 min with 1 *μ*M [^3^H]-choline (specific activity 2.5 *μ*Ci nmol^−1^) under electrical field stimulation (1 Hz-frequency, 1 ms pulse width). Following loading, the washout Tyrode's solution contained hemicholinium-3 (10 *μ*M) to prevent choline uptake. After a 60 min period of washout, bath samples (3 mL) were automatically collected every 3 min using a fraction collector (Gilson, FC203B, France). Tritium content of the samples was measured by liquid scintillation spectrometry (% counting efficiency: 40 ± 2%).

Test drugs were added 15 min before* S*_2_. The change in the ratio between the evoked [^3^H]-ACh release during the two stimulation periods (*S*_2_/*S*_1_) relative to that observed in control situations (in the absence of test drugs) was taken as a measure of the effect of the tested drugs. None of the drugs changed significantly (*P* > 0.05) basal tritium outflow.

### 2.5. Kinetic Experiments of the Extracellular Catabolism of Purines and HPLC Analysis

For the kinetic experiments of the extracellular catabolism of adenine nucleotides and adenosine, strips from the longitudinal muscle with the myenteric plexus attached (LM-MP) were mounted in a 2 mL organ bath. All experiments were performed at 37°C. Preparations were superfused with gassed (95% O_2_ and 5% CO_2_) Tyrode's solution containing (mM): NaCl 137, KCl 2.7, CaCl_2_ 1.8, MgCl_2_ 1, NaH_2_PO_4_ 0.4, NaHCO_3_ 11.9, and glucose 11.2. After a 30 min equilibration period, the preparations were incubated for 45 min with Tyrode's solution to eliminate endogenous interfering compounds. Following a washout period of 10 min, the preparations were then incubated with 30 *μ*M of ATP, ADP, AMP, or adenosine (zero time). Samples of 75 *μ*L were collected from the organ bath at different times up to 45 min for HPLC (with UV detection) analysis of the variation of substrate disappearance and product formation ([12, 18, 19]; see also [31]). The concentrations of the substrate and products were plotted as a function of time (progress curves). In some experiments, we measured unbound 5′-nucleotidase and adenosine deaminase activities in the fluid retained in the bath after removing the LM-MP preparation. We followed a similar experimental protocol to that used for studying the kinetics of the extracellular catabolism of adenine nucleotides and adenosine in the presence of the preparations.

### 2.6. Release of ATP and Adenine Nucleosides (Adenosine Plus Inosine)

Experiments were performed using an automated perfusion system for sample collecting for given time periods, therefore improving the efficacy of HPLC (with diode array detection) and bioluminescence analysis. After the 30 min equilibration period, the preparations were incubated with 1.8 mL gassed Tyrode's solution, which was automatically changed every 15 min by emptying and refilling the organ bath with the solution in use. The preparations were electrically stimulated once, 15 min after starting sample collection (zero time), using 3000 square wave pulses of 1 ms duration delivered at a 5 Hz frequency. In these experiments, only the sample collected before stimulus application and the two samples collected after stimulation were retained for analysis. Bath aliquots (50–250 *μ*L) were frozen in liquid nitrogen immediately after collection, stored at −20°C (the enzymes are stable for at least 4 weeks), and analysed within 1 week of collection by HPLC with diode array detection (Finigan Thermo Fisher Scientific System LC/DAD, equipped with an Accela Pump coupled to an Accela Autosample, a diode array detector and an Accela PDA running the X-Calibur software chromatography manager). Chromatographic separation was carried out through a Hypersil GOLD C18 column (5 *μ*M, 2.1 mm × 150 mm) equipped with a guard column (5 *μ*m, 2.1 mm × 1 mm) using an elution gradient composed of ammonium acetate (5 mM, with a pH of 6 adjusted with acetic acid) and methanol. During the procedure the flow rate was set at 200 *μ*L/min and the column temperature was maintained at 20°C. The autosampler was set at 4°C and 50 *μ*L of standard or sample solution was injected, in duplicate, for each HPLC analysis. In order to obtain chromatograms and quantitative analysis with maximal sensibility, the diode array detection wavelength was set at 259 nm for adenosine and 248 nm for inosine. In parallel, the ATP content of the same samples was evaluated with the luciferin-luciferase ATP bioluminescence assay kit HS II (Roche Applied Science, Indianapolis, Indiana) (see e.g., [31, 32]). Luminescence was determined using a multidetection microplate reader (Synergy HT, BioTek Instruments). Stimulation-evoked release of adenine nucleotides and nucleosides was calculated by subtracting the basal release, measured in the sample collected before stimulation, from the total release of adenine nucleotides and nucleosides determined after stimulus application.

### 2.7. Immunofluorescence Staining and Confocal Microscopy Observation

LM-MP fragments were isolated from the rat ileum as previously described. The LM-MP fragments were stretched to all directions and pinned onto Petri dishes coated with Sylgard. The tissues, then, were fixed in PLP solution (paraformaldehyde 2%, lysine 0.075 M, sodium phosphate 0.037 M, sodium periodate 0.01 M) for 16 h at 4°C. Following fixation, the preparations were washed three times for 10 min each using 0.1 M phosphate buffer. At the end of the washout period, tissues were cryoprotected during 16 h with a solution containing anhydrous glycerol 20% and phosphate buffer 0.1 M at 4°C and, then, stored at −20°C for further processing. Once defrosted, tissue fragments were washed with tamponated phosphate saline buffer (PBS) and incubated with a blocking buffer, consisting in foetal bovine serum 10%, bovine serum albumin 1%, triton X-100 0.3% in PBS, for 2 h; washout was facilitated by constant stirring of the samples. After blocking and permeabilization, samples were incubated with selected primary antibodies (see Table 1) diluted in the incubation buffer (foetal bovine serum 5%, serum albumin 1%, Triton X-100 0.3% in PBS), at 4°C, for 48 h. For double immunostaining, antibodies were combined before application to tissue samples. Following the washout of primary antibodies with PBS supplemented with triton X 0.3% (3 cycles of 10 min), tissue samples were incubated with species-specific secondary antibodies in the dark for two hours, at room temperature. Finally, tissue samples were mounted on optical-quality glass slides using VectaShield as mounting media (VectorLabs) and stored at 4°C. Observations were performed and analyzed with a laser-scanning confocal microscope (Olympus FluoView, FV1000, Tokyo, Japan).

### 2.8. Antibody Production

All primary antibodies used in this study have previously been validated [33–37]: rabbit rN1-6L to rat NTPDase 1, rabbit rN2-6L to rat NTPDase2, rabbit rN3-1L to rat NTPDase3, guinea pig rN8-8C to rat NTPDase8, rabbit rNU-9L, and rabbit rNU-4L to rat ecto-5′-nucleotidase/CD73. Genetic immunization protocol was carried out with plasmids (pcDNA3.1) encoding each protein, in New Zealand rabbits for antibodies against rat NTPDase1, rat NTPDase2, rat NTPDase3, rat ecto-5′-nucleotidase, and Hartley guinea pigs for rat NTPDase8 antibodies. All procedures were approved by the Canadian Council on Animal Care and the Université Laval Animal Welfare Committee.

### 2.9. Materials and Solutions

Adenosine 5′-triphosphate (ATP), adenosine 5′-diphosphate (ADP), adenosine 5′-monophosphate (AMP), adenosine, inosine, hypoxanthine; 2,4,6-trinitrobenzenesulphonic acid (TNBS); choline chloride, paraformaldehyde (prills), lysine, sodium periodate, anhydrous glycerol, fetal bovine serum (Sigma, St Louis, MO, USA); serum albumin, triton X-100, metanol, potassium dihydrogen phosphate (KH_2_PO_4_) (Merck, Darmstadt, Germany); dipyridamole (Boehringer Ingelheim, Germany); 1,3-dipropyl-8-cyclopentylxanthine (DPCPX) (Research Biochemicals, Natick, MA, USA); mibefradil dihydrochloride, tetrodotoxin (TTX), 4-(-2-[7-amino-2-{2-furyl}{1,2,4}triazolo{2,3-a}{1,3,5}triazin-5-yl-amino]ethyl)phenol (ZM 241385) (Tocris Cookson Inc., UK); [methyl-^3^H]-choline chloride (ethanol solution, 80 Ci mmol^−1^) (Amersham, UK); PicA reagent (Waters corporation, Milford, USA); ATP bioluminescence assay kit HS II (Roche Applied Science, Indianapolis, Indiana); medetomidine hydrochloride (Domitor, Pfizer Animal Health); atipamezole hydrochloride (Antisedan, Orion, Espoo, Finland); ketamine hydrochloride (Imalgene, Merial, Lyon, France); Sodium chloride 0.9%, tramadol hydrochloride (Labesfal, Santiago de Besteiros, Portugal).

All drugs were prepared in distilled water. All stock solutions were stored as frozen aliquots at −20°C. Dilutions of these stock solutions were made daily and appropriate solvent controls were done. No statistically significant differences between control experiments, made in the absence or in the presence of the solvents at the maximal concentrations used (0.5% v/v), were observed. The pH of the perfusion solution did not change by the addition of the drugs in the maximum concentrations applied to the preparations.

### 2.10. Presentation of Data and Statistical Analysis

The values are expressed as mean ± SEM, with *n* indicating the number of animals used for a particular set of experiments. Statistical analysis of data was carried out using paired or unpaired Student's* t*-test or one-way analysis of variance (ANOVA) followed by Dunnett's modified* t*-test. *P* < 0.05 represents significant differences.

## 3. Results

### 3.1. Postinflammatory Ileitis Led to Increases in Gastrointestinal Transit in the Rat

**Figure 1:**
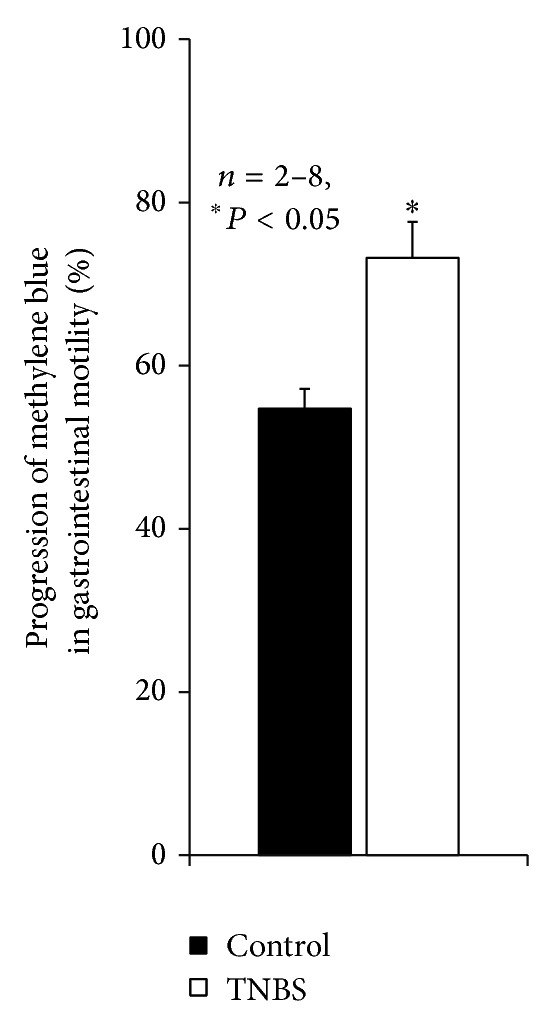
Progression of methylene blue dye gavage along the gastrointestinal tract during 30 minutes in control and TNBS-injected animals. The total length of the small intestine was used to normalize data. The vertical bars represent SEM. *P < 0.05 (unpaired Student's* t*-test) represents significant differences as compared to the control situation.

Histology sections of the ileum of TNBS-injected rats were characterized using the criteria proposed by Pontell et al. [3] and Jergens [26] (see Section 2). One week after the TNBS treatment we observed a proliferative regeneration of the mucosal integrity. Postinflammatory infiltrates consisting mainly of eosinophils, lymphocytes, and macrophages were scarcely seen but when present extended to the submucosa, enteric plexuses, and muscular layers. At that time, the total ileal wall thickness of TNBS-treated rats was increased due to increase in thickness of both mucosal and muscular layers. The gastrointestinal motility was evaluated prior to sacrifice of the animals. This was done by measuring the progression of methylene blue dye gavage during 30 min.  Figure 1 shows that methylene blue dye progression was significantly (*P* < 0.05) faster in TNBS-injected animals than in control animals, indicating that GI propulsion is increased in postinflammatory ileitis. Although TNBS-ileitis in the rat lacks a typical chronic phase, the postinflammatory phase is characterized by increases in motility (see e.g. [38]).

### 3.2. Adenosine Neuromodulation Is Impaired in Postinflammation Ileitis

**Figure 2:**
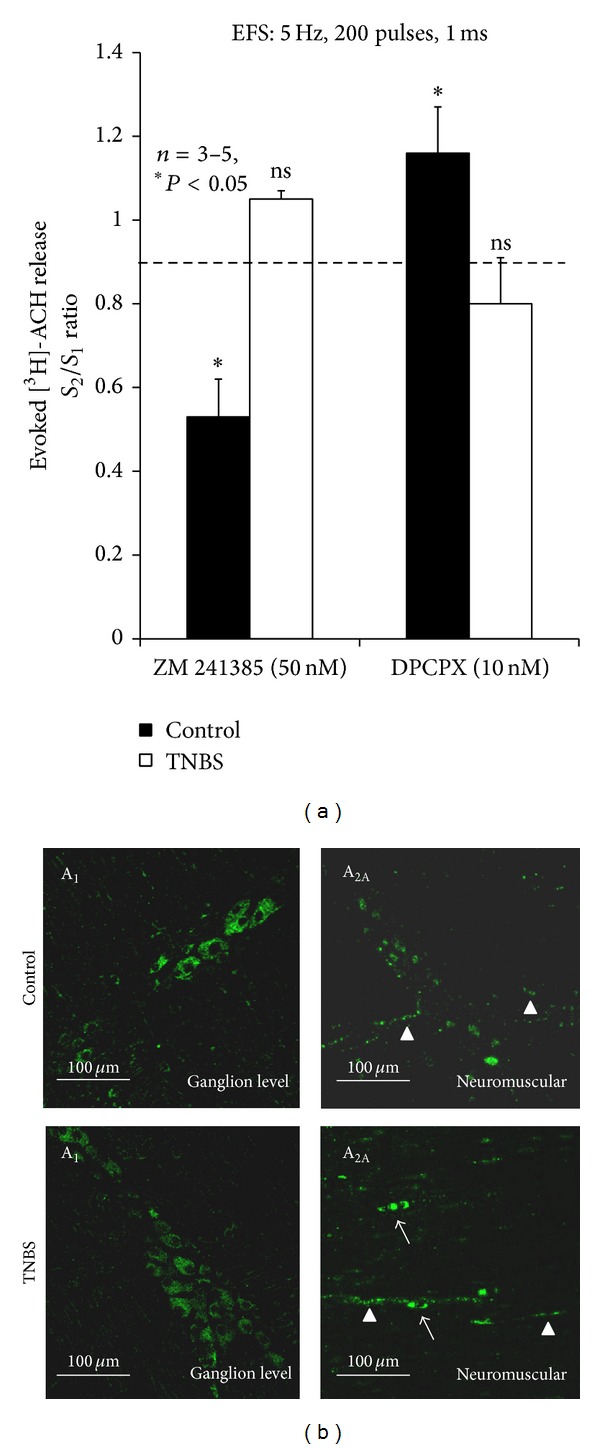
(a) Actions of selective adenosine A_1_ and A_2A_ receptor antagonists on [^3^H]-ACh release from myenteric motoneurons stimulated electrically (5 Hz, 200 pulses, 1 ms duration). DPCPX (10 nM, A_1_ receptor antagonist) and ZM 241385 (50 nM, A_2A_ receptor antagonist) were added to the incubation media before the second period of stimulation (*S*_2_) and were present throughout the assay. The ordinates are changes in* S*_2_/*S*_1_ ratios compared to the* S*_2_/*S*_1_ ratio obtained in control conditions, that is, with no drugs added. The data are means ± SEM of three to five individual experiments. *P < 0.05 (one-way ANOVA followed by Dunnett's modified* t*-test) represent significant differences when compared to the situation where no drugs were added (dashed line). (b) Confocal images of whole-mount preparations of longitudinal muscle-myenteric plexus preparations of the ileum from control and TNBS-injected rats. Adenosine A_1_ receptor immunoreactivity is predominantly present in ganglion neuronal cell bodies in control and TNBS-injected preparations. Adenosine A_2A_ receptor imunoreactivity is mainly present in nerve bundles and axon terminals of myenteric neurons (triangles). In inflamed preparations, adenosine A_2A_ receptor staining is also observed in few mononuclear cells infiltrating the neuromuscular layer (arrows).

Previous reports suggested that the purinergic control of cholinergic nerve activity may be significantly impaired during chronic intestinal inflammation, both in the colon of rabbits and guinea-pigs [39, 40], and in mice ileum [14]. Despite the differences in the mechanisms used to produce intestinal inflammation among these groups, all are unanimous on the indication for the need of more studies in order to investigate the underlying pathways responsible for disturbed purinergic neuromodulation. Hence, we focused our attention on the net tonic action of endogenous adenosine on electrically evoked [^3^H]-ACh release from myenteric motoneurons seven days after an inflammatory insult to the rat ileum. In preparations from healthy rats, the A_1_ receptor antagonist, DPCPX (10 nM) increased the release of [^3^H]-ACh by 27 ± 4% (*n* = 4) (Figure 2(a)). Conversely, selective blockade of adenosine A_2A_ receptors with ZM 241385 (50 nM) significantly decreased the evoked tritium outflow by 37 ± 10% (*n* = 6). The results indicate that endogenous adenosine exerts a dual role on evoked [^3^H]-ACh release via the activation of inhibitory A_1_ and excitatory A_2A_ receptors in the rat ileum (cf. [18, 19]). However, when similar experiments were conducted in the LM-MP seven days after ileal inflammation none of the adenosine receptor antagonists caused any measurable change on evoked transmitter release (Figure 2(a)). These results indicate that adenosine modulation of cholinergic nerve activity is severely impaired in postinflammation ileitis, as previously suggested using both myographic recordings and electrophysiology methods (see above).

Several authors hypothesized that purinoceptors downregulation in the enteric nervous system can occur during prolonged contact with purines. This was predicted since purines, such as ATP and adenosine, can be released from activated inflammatory cells [22] in the vicinity of myenteric neurons [24]. To elucidate this contention, we performed immunolocalization studies by confocal microscopy to assess changes in the expression and localization of both A_1_ and A_2A_ adenosine receptors that could contribute to explaining the lack of endogenous adenosine neuromodulatory tonus in the inflamed ileum. The immunoreactivity against A_1_ receptors is located predominantly on cell bodies of myenteric neurons of the ileum in control as well as in TNBS-injected rats (Figure 2(b)). The longitudinal muscle fibers also stain positively with the adenosine A_1_ receptor antibody (data not shown). Yet, no significant differences were observed between control and inflamed preparations (Figure 2(b)). Likewise, the A_2A_ receptor immunoreactivity, which is more evident on myenteric nerve terminals, also did not significantly differ among control and inflamed tissues (Figure 2(b)). Interestingly, we observed staining against the A_2A_ receptor in few mononuclear cells (probably lymphocytes) infiltrating the neuromuscular level, which might be responsible for the immunosuppressant effect of A_2A_ receptors activation in experimental ileitis [41, 42]. Mononuclear infiltrates containing A_2A_-positive immune cells may contribute to the increased mRNA expression of this receptor found in chronic inflamed intestinal tissues [43]. Although we cannot exclude at this stage differences in the intracellular signaling pathway triggered by activation of the two high affinity adenosine receptor subtypes, A_1_ and A_2A_, in the LM-MP of the rat ileum, our data suggest that receptors expression is fairly conserved in the ileum seven days following an inflammatory insult and, apparently, does not underscore the lack of adenosine neuromodulation tonus in this condition (see, e.g., [14, 43, 44]).

### 3.3. Postinflammatory Ileum Releases More ATP, Which Is Not Followed by Adenosine Formation

**Figure 3:**
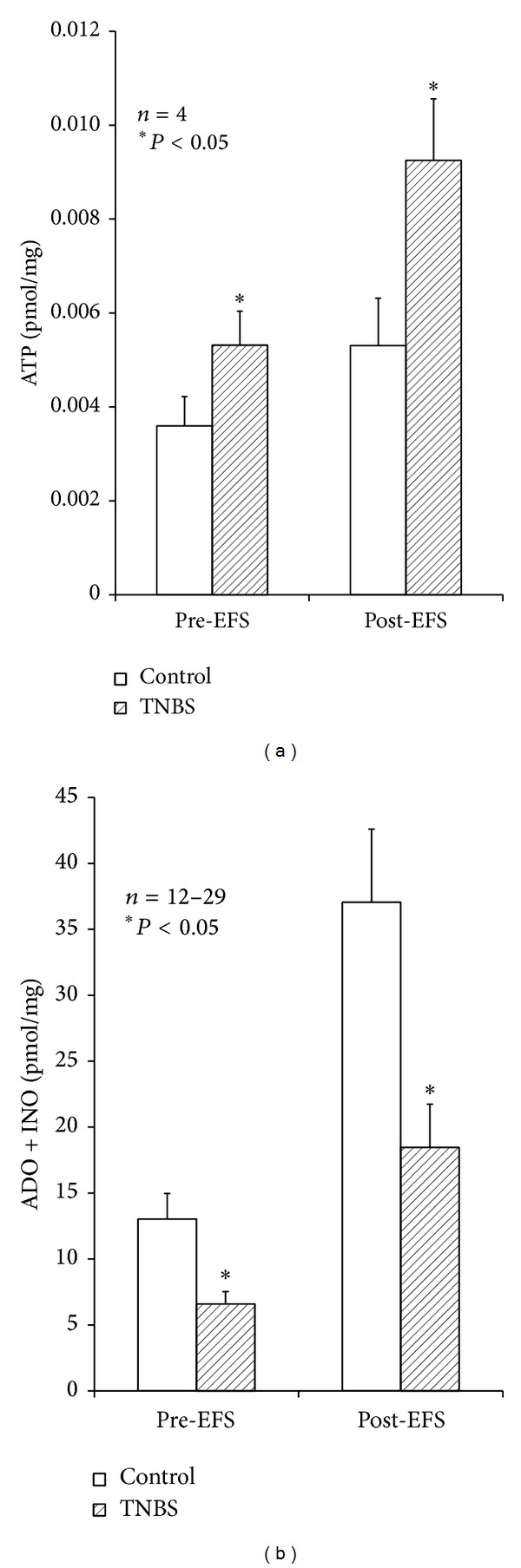
Effect of electrical field stimulation (EFS) on extracellular accumulation of purines in the LM-MP of control and inflamed rat ileum. The preparations were incubated with Tyrode's solution for 15 min and then subject to EFS (5 Hz frequency, 3000 pulses of 1 ms duration). Samples collected from the incubation media were analysed by HPLC to separate and quantify purine nucleosides; ATP outflow was assessed using the luciferin-luciferase bioluminescence assay. Illustrated are ATP (a) and purine nucleosides (b) content of bath samples collected immediately before (Pre-EFS) and after (Post-EFS) LM-MP stimulation expressed in pmol/mg of tissue. Data are means ± SEM of an *n* number of individual experiments. *P < 0.05 (one-way ANOVA followed by Dunnett's modified* t*-test) represent significant differences as compared to control animals.

It has been hypothesized, but not yet proven, that disruption in ATP release and/or breakdown are most likely explanations for the suppression of purinergic neuromuscular transmission in inflamed regions of the distal colon [40]. It is possible that inflammatory mediators affect mitochondrial function, and therefore ATP synthesis, in the ulcerated region of the chronic inflamed intestine. Another possibility is that there may be increased expression of ectonucleotidases, which are responsible for the breakdown of ATP [12, 45], as has been demonstrated in the purinergic sympathetic regulation of submucosal blood vessels [45]. Here, we measured the extracellular accumulation of ATP (by bioluminescence) in parallel with adenosine plus inosine content (by HPLC with diode array detection) in samples collected immediately before and after electrical stimulation of LM-MP preparations of the ileum of both control and TNBS-injected rats.  Figure 3 shows that, in healthy animals, stimulation of the LM-MP at a frequency of 5 Hz (3000 pulses of 1 ms duration) yields increased amounts of ATP in the incubation fluid, which was followed by even higher extracellular accumulation of adenine nucleosides, consisting mostly of adenosine and inosine. This pattern was totally reversed seven days after inflammation of the rat ileum. Basal levels of ATP significantly (*P* < 0.05) increase (Figure 3(a)), whereas the baseline of endogenous adenosine plus inosine decreased (Figure 3(b)), in the fluid collected from TNBS-treated LM-MP of the rat ileum as compared to control tissues. Although the amount of ATP and adenine nucleosides increased from baseline following electric stimulation of LM-MP preparations in both animal groups, released ATP levels reached higher values in inflamed preparations than in controls (Figure 3(a)), but the opposite was observed regarding the content of adenosine plus inosine (Figure 3(b)). These results indicate that, in contrast to previous hypothesis, the amount of ATP released in basal conditions and following electric stimulation of TNBS-treated ileal preparations was significantly higher than in control tissues, although one could not discard at this stage that ATP accumulation could also result from a decrease in the extracellular breakdown of the nucleotide (see below). Despite this increase in ATP accumulation, the content of adenosine plus inosine in the superfusates from TNBS-treated animals was severely decreased, which might contribute to explaining the lack of endogenous adenosine neuromodulatory tonus in postinflammatory ileitis (Figure 2; see also [43]).

**Figure 4:**
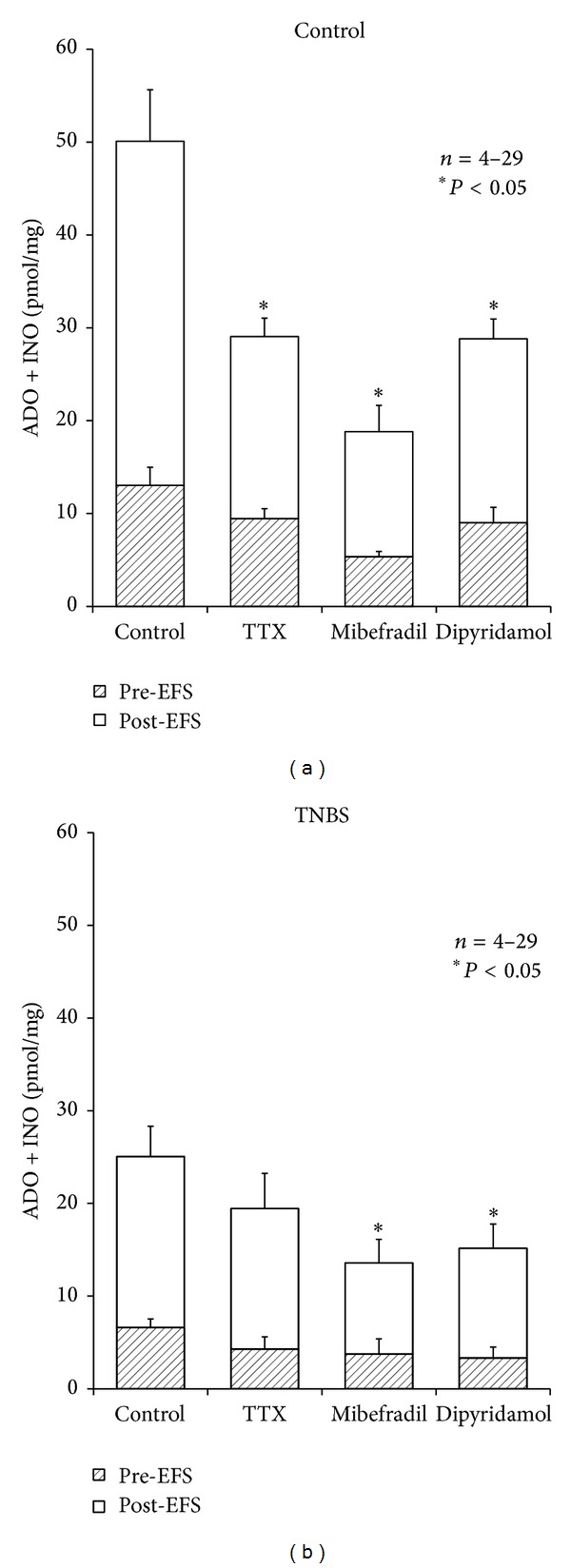
Influence of tetrodotoxin (TTX, 1 *μ*M), mibefradil (3 *μ*M), and dipyridamole (0.5 *μ*M) on stimulation-evoked release of purine nucleosides (adenosine plus inosine) from the LM-MP of control and TNBS-injected rat ileum. Drugs were in contact with the preparations for at least 15 min before stimulus application (5 Hz frequency, 3000 pulses of 1 ms duration). Samples collected from the incubation media were analysed by HPLC with diode array detection. Illustrated is the purine nucleosides content of bath samples collected immediately before (Pre-EFS) and after (Post-EFS) stimulation of LM-MP expressed in pmol/mg of tissue. Data represent the means ± SEM of an *n* number of individual experiments. *P < 0.05 (one-way ANOVA followed by Dunnett's modified* t*-test) represents significant differences from the evoked amount of purine nucleosides detected in control conditions where no drugs were added to the preparations.

In healthy tissue, but not in the ileum following the inflammatory insult, stimulus-evoked adenosine release was partially dependent on neuronal activity. This was evidenced because pretreatment of the preparations with tetrodotoxin (TTX), applied in a concentration (1 *μ*M) that essentially abolished evoked [^3^H]-ACh release, reduced by about 50% adenine nucleosides outflow from control tissues with almost no effect in TNBS-treated preparations (Figure 4). These findings indicate that barely half of the extracellular adenosine released from stimulated LM-MP preparations from healthy rats comes from TTX-sensitive neuronal cells, but this source may be severely affected in the postinflammation phase of TNBS-ileitis due to neuronal dysfunction, as demonstrated by several authors. Under conditions leading to myenteric neuronal dysfunction such as chronic inflammation, ATP release may be shifted from a neuronal origin towards other cell sources (e.g., smooth muscle fibers, glial cells, and interstitial cells of Cajal, infiltrating immune cells) (see, e.g., [7]). As a matter of fact, evoked ATP release from TTX-resistant nonneuronal sources increased (*P* < 0.05) above baseline from 7.4 ± 0.6 fmol/mg (*n* = 4) in control animals to 13.2 ± 0.2 fmol/mg (*n* = 4) in TNBS-treated preparations.

### 3.4. Smaller Amounts of Adenosine Released from Inflamed Ileum Originate from Activated Pacemaker Interstitial Cells of Cajal via Equilibrative Nucleoside Transporters

Considering that adenosine may also be released as such from nonneuronal cells at the tripartite myenteric synapse, we tested whether stimulus-evoked smooth muscle contraction and activation of pacemaker interstitial cells of Cajal (ICCs) affected the outflow of adenine nucleosides from the ileum of control and TNBS-injected rats. Blockade of smooth muscle contractions with the Ca_v_1 (L-type) voltage-sensitive calcium channel inhibitor, nifedipine (1–5 *μ*M), did not affect the accumulation of adenine nucleosides in bath samples collected immediately before and after electric field stimulation to the LM-MP from healthy animals [19]. However, selective blockade of Ca_v_3 (T-type) calcium channels located predominantly in ICCs with mibefradil (3 *μ*M) significantly (*P* < 0.05) depressed the outflow of adenine nucleosides from both control and TNBS-treated preparations (Figure 4). The results indicate that ICCs are the main source of adenosine released from stimulated LM-MP of the ileum in the postinflammation phase. Data also indicate that ICCs cooperate with myenteric neurons to increase extracellular adenosine in preparations from healthy rats.

Given that adenosine is neither stored nor released as a classical neurotransmitter and previous findings from our laboratory demonstrated that the ectonucleotidase pathway contributes only partially to the total interstitial adenosine concentration in the myenteric plexus [19], we tested the involvement of equilibrative nucleoside transporters to extracellular adenosine released from tissues isolated from control and TNBS-injected rats. The nucleoside transport inhibitor, dipyridamole (0.5 *μ*M), decreased proportionally, and by a similar magnitude to that obtained with mibefradil (3 *μ*M) the outflow of adenine nucleosides from both control and inflamed tissues (Figure 4). Thus, it is likely that extracellular adenosine detected in ileum following an inflammatory insult originates predominantly from activated interstitial cells of Cajal via equilibrative nucleoside transporters.

Another potential source of endogenous adenosine in the rat ileum may be adenosine 3′,5′-cyclic monophosphate (cAMP) extruded from activated cells, which may be converted to AMP and then to adenosine by ecto-phosphodiesterase and ecto-5′-nucleotidase/CD73, respectively [46]. Despite the existence of a functional extracellular cAMP-adenosine pathway in the rat ileum, we were unable to detect any measurable amounts of cAMP by HPLC analysis (with diode array detection) in the samples used to quantify adenine nucleosides, even though we could identify a chromatographic peak corresponding to cAMP spectrum but with a higher retention time than adenosine using a 30-*μ*M external standard (data not shown). These results suggest that, under the present experimental conditions, the cAMP-adenosine pathway does not account significantly for endogenous adenosine formation in the LM-MP of the rat ileum.

### 3.5. The Kinetics of the Extracellular Catabolism of Adenine Nucleotides (ATP and ADP) Was Not Different in Control and Postinflammation Preparations of the Rat Ileum

**Figure 5:**
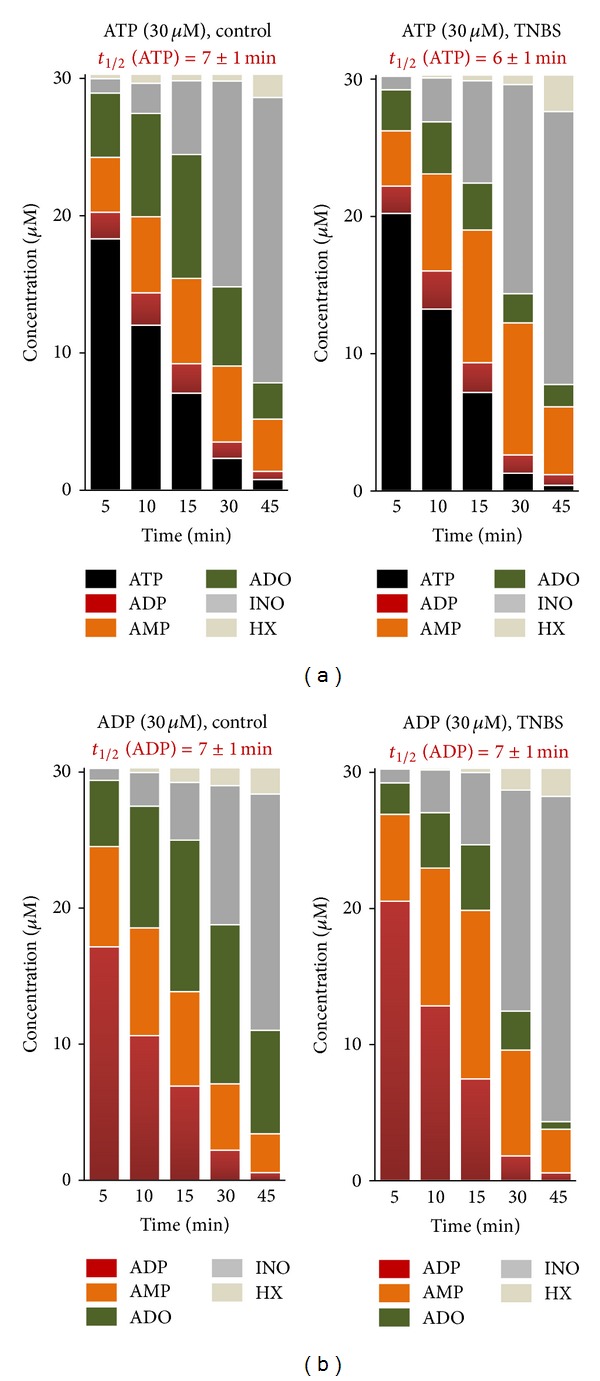
Kinetics of the extracellular catabolism of ATP (a) and ADP (b) in the LM-MP of the ileum of control and TNBS-injected rats. ATP and ADP (30 *μ*M) were incubated at zero time (see Section 2). Collected samples were analysed by HPLC with UV detection to separate and quantify ATP (black), ADP (red), AMP (orange), ADO (green), INO (dark grey), and HX (light grey). Average results obtained in four experiments.

Despite the observation that the ileum in the postinflammation phase releases more ATP than control preparations, we observed a decrease in the extracellular adenosine content in samples collected both under basal and stimulated conditions. It is, therefore, possible that disruption of ATP catabolism into adenosine via ecto-NTPDases may occur in the inflamed LM-MP of the ileum.  Figure 5 illustrates the time course of the extracellular catabolism of ATP and ADP in the LM-MP of the rat ileum from control and TNBS-treated rats. No significant differences (*P* > 0.05) were observed among the half-degradation times of ATP (30 *μ*M) and ADP (30 *μ*M) in control and TNBS-treated preparations, that is, the half degradation time of the two adenine nucleotides ranged from 6 to 7 min independently of the experimental condition. Extracellular ATP (30 *μ*M) was metabolized into ADP, AMP, adenosine, inosine, and hypoxanthine, whose concentrations increased with time. Higher amounts of AMP as compared to ADP were detected in the bath at all time points following ATP (30 *μ*M) application (Figure 5(a)). ADP (30 *μ*M) catabolism led to AMP, adenosine, inosine, and hypoxanthine (Figure 5(b)). Interestingly, we detected differences in the accumulation of AMP and its degradation product, adenosine, between control and postinflamed preparations incubated either with ATP (30 *μ*M) or ADP (30 *μ*M). The extracellular AMP content increased (orange bars), whereas adenosine decreased (green bars), proportionally in the incubation fluid of TNBS-treated preparations as compared to those isolated from control rats following incubation with either ATP (30 *μ*M) or ADP (30 *μ*M) (Figure 5). Increases in AMP content of bath samples from postinflammation ileum reached the control levels 45 min after starting incubation with either ATP (30 *μ*M) or ADP (30 *μ*M), that is, when the concentration of adenine nucleotides reached minimum (Figure 5).

### 3.6. Feed-Forward Inhibition of Ecto-5′-Nucleotidase/CD73 by Adenine Nucleotides Controls Adenosine Formation in Postinflammation Ileitis

**Figure 6:**
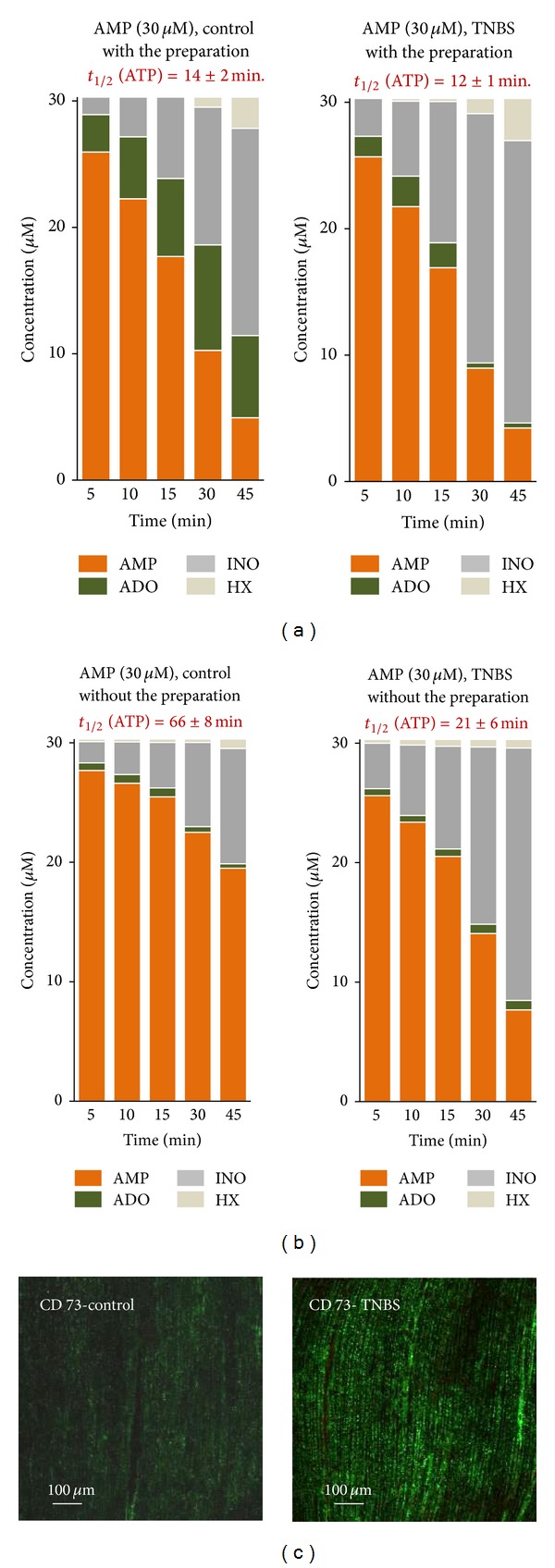
Kinetics of the extracellular catabolism AMP (30 *μ*M) in the LM-MP of the ileum of control and TNBS-injected rats (a). The residual ecto-5′-nucleotidase/CD73 activity (soluble form) in the incubation fluid after removing the preparations from the organ bath, is also shown (b). AMP (30 *μ*M) was incubated at zero time (see Section 2). Collected samples were analysed by HPLC with UV detection to separate and quantify AMP (orange), ADO (green), INO (dark grey) and HX (light grey). Average results obtained in four experiments. Panel (c) shows that immunoreactivity against ecto-5′-nucleotidase/CD73 in longitudinal smooth muscle fibers of the ileum from TNBS-treated rats is much more evident than in control animals.

Data from Figure 5 clearly indicate that differences in adenosine formation from the extracellular catabolism of adenine nucleotides in postinflammation ileitis implicate AMP dephosphorylation by ecto-5′-nucleotidase, which is the rate limiting enzyme for adenosine formation in the rat myenteric plexus [12]. Surprisingly, we did not observe significant changes (*P* > 0.05) in the half degradation time of AMP (30 *μ*M) between control and TNBS-treated preparations (Figure 6(a)). If any difference has to be mentioned, is the fact that the inflamed ileum accumulated proportionally less adenosine in the extracellular milieu as compared to control tissues when AMP (30 *μ*M) was used as substrate (Figure 6(a)). Overall, the results indicate that adenosine is being rapidly converted into inosine by ADA in the extracellular space of both control and TNBS-treated preparations (see below), given that stoichiometry of AMP conversion into adenosine, inosine and hypoxanthine is kept unaltered ruling out a significant contribution of the nucleoside uptake system (compared to [18]).

Since ecto-5′-nucleotidase/CD73 may be cleaved from its membrane GPI anchor by proteolysis retaining its catalytic activity in the incubation fluid, we decided to test whether binding of this enzyme to the plasma membrane was somehow affected during chronic inflammation in order to explain the differences detected in adenosine formation from the hydrolysis of adenine nucleotides. To this end, we evaluated the 5′-nucleotidase activity in the fluid remaining in the bath after removing the preparation following a 45-min incubation period (Figure 6(b)). Under these experimental conditions, the half-life of AMP (30 *μ*M) was significantly (*P* < 0.05) decreased in TNBS-treated preparations (*t*_1/2_ = 21 ± 6 min, two-times slower than with the tissue present) as compared to controls (*t*_1/2_ = 66 ± 8 min, almost five-times slower than with the tissue present). Data suggest that postinflammation ileitis causes an increase in unbound soluble forms of 5′-nucleotidase, which contribute to dephosphorylation of extracellular AMP into adenosine without changing the global enzyme activity in the tissue. Notwithstanding, functional repercussions may be expected from this fact taking into consideration that ecto-5′-nucleotidase/CD73 is concentrated in the myenteric smooth muscle cell layer of healthy ileum [7], whereas unbound soluble forms of the enzyme may generate adenosine from extracellular AMP way from the most common site at the myenteric neuromuscular synapse in the inflamed ileum.

**Figure 7:**
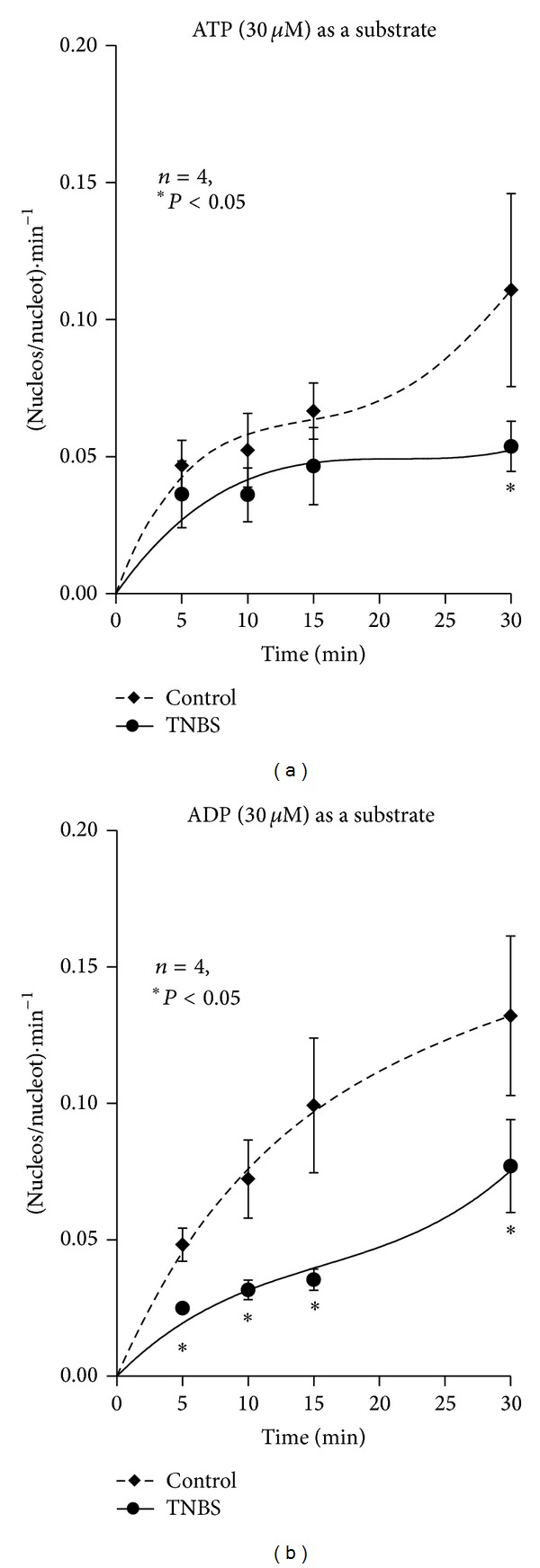
Activity of ecto-5′-nucleotidase/CD73 when ATP (a) and ADP (b) were used as substrates of the ectonucleotidase cascade in the LM-MP of the ileum of control and TNBS-injected rats. ATP and ADP (30 *μ*M) were incubated at zero time. Ecto-5′-nucleotidase/CD73 activity was evaluated by quantifying the ratio [Nucleosides] : [Nucleotides]/min. Average results obtained in four experiments; the vertical bars represent the SEM and are shown when they exceed the symbols in size. *P < 0.05 (one-way ANOVA followed by Dunnett's modified* t*-test) represents significant differences as compared to control animals.

Given that we did not observe any significant differences in the AMP catabolism between control and TNBS-treated preparations (see Figure 6(a)) and we, still, detected increases in ecto-5′-nucleotidase/CD73 immunoreactivity in the longitudinal neuromuscular layer of the inflamed rat ileum (Figure 6(c); see also [43]), we hypothesized that ecto-5′-nucleotidase/CD73 could be feed-forwardly inhibited by high extracellular levels of ATP and/or ADP which bind to the catalytic site of the enzyme blunting adenosine formation from AMP in inflamed preparations (Figure 3; see also [47]). The ecto-5′-nucleotidase activity was evaluated by quantifying the ratio [Nucleosides] : [Total nucleotides] per min, which is a direct measure of the activity of ecto-5′-nucleotidase, using either ATP (30 *μ*M) or ADP (30 *μ*M) as substrates.  Figure 7(a) shows that this ratio increased progressively with time upon consumption of ATP or ADP in the incubation media. The activity of ecto-5′nucleotidase/CD73 was significantly (*P* < 0.05) impaired in the LM-MP of the ileum following an inflammatory insult, particularly when ADP (30 *μ*M) was used as substrate. These findings agree with data from Figure 5, showing that adenosine formation is delayed with a compensatory increase in AMP accumulation in the inflamed ileum as compared to control preparations which is compatible with our hypothesis that ATP and/or ADP feed-forwardly inhibit ecto-5′-nucleotidase/CD73 in postinflammation ileitis.

### 3.7. Tissue Distribution Changes of NTPDase2, NTPDase3, and NTPDase8 in Postinflammation Ileitis

**Figure 8:**
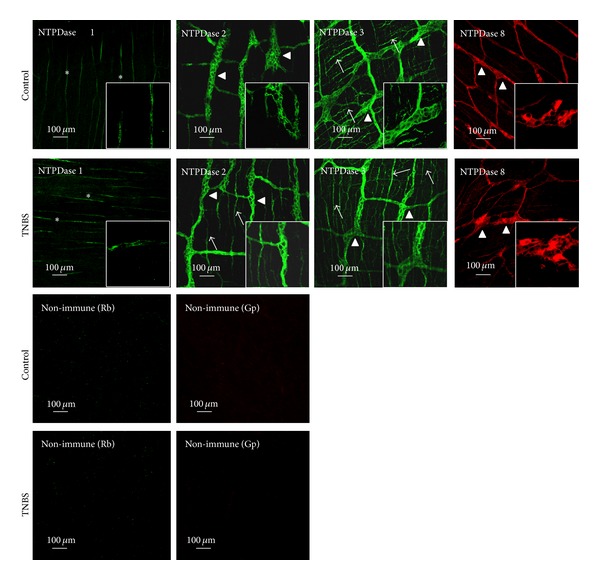
Localization of NTPDase1, NTPDase2, NTPDase3, and NTPDase8 immunoreactivity in single confocal images of whole-mount preparations of the longitudinal muscle-myenteric plexus of the ileum of control and TNBS-injected rats. In healthy animals, NTPDase 1 immunoreactivity is present only in blood vessels (asterisks); NTPDase2 immunoreactivity is present predominantly in ganglion neuronal cell bodies (triangles) and large ramifications (primary meshwork) of the myenteric plexus, whereas NTPDase3 is also evident on myenteric axon terminals (arrows). In TNBS-treated preparations the NTPDase2 staining acquires a pattern that is very similar to that of NTPDase3, with NTPDase2 immunoreactivity also appearing in nerve bundles and axon terminals of myenteric neurons. NTPDase8 stains few ganglion neuronal cell bodies and large ramifications (primary meshwork) of the myenteric plexus; this pattern did not significantly change among control and TNBS-treated animals. No staining was obtained when nonimmune sera from host species (rabbit, Rb, and guinea-pig, Gp) were used instead of interest primary antibodies.

The relative amounts of ATP and/or ADP near the ecto-5′-nucleotidase/CD73, which is concentrated in the myenteric smooth muscle layer ([7]; see also Figure 6(c)), are paramount to predict the magnitude of feed-forward inhibition of the enzyme by released adenine nucleotides. This may be determined by the expression and colocalization of NTPDases responsible for the kinetics of the catabolism of adenine nucleotides in control as well as in inflamed ileum.  Figure 8 shows that in control LM-MP preparations the immunoreactivity against NTPDase2 is restricted to ganglion cell bodies and large ramifications (primary meshwork) of the myenteric plexus (compared to [48]). This contrasts with the localization of NTPDase3 immunoreactivity, which is also evident on myenteric nerve trunks and terminals corresponding to secondary and tertiary neuronal meshworks, respectively [49]. At this time, we cannot exclude the presence of this enzyme on intramuscular ICCs. This pattern changes in the postinflammation ileum. In TNBS-treated preparations, the NTPDase2 immunoreactivity is also observed in the secondary and tertiary neuronal meshwork, meaning that ADP formation from released ATP by stimulated enteric neurons [6] and smooth muscle fibres [7] may contribute to prolong feed-forward inhibition of muscle-bound ecto-5′-nucleotidase/CD73 at the myenteric neuromuscular synapse following an inflammatory insult. Apparently no significant differences were found in the expression and localization of NTPDase3 in the inflamed LM-MP of the rat ileum. Regarding NTPDase8, we were able to demonstrate its presence in few neuronal cell bodies of myenteric ganglia, with much lesser expression at the neuromuscular layer (Figure 8). Phenotypic characterization of NTPDase8 positive neuronal cell bodies deserves future investigations. Like NTPDase3, we found no gross changes in NTPDase8 immunoreactivity between control and TNBS-treated animals. We focused our attention on these three ectoenzymes, because we detected no immunoreactivity against NTPDase1 in the LM-MP of the rat ileum besides few blood vessels (Figure 8).

### 3.8. Postinflammation Ileitis Is Accompanied by Higher Adenosine Deaminase (ADA) Activity: Contribution of Membrane-Bound (ecto-ADA) and Soluble (exo-ADA) Forms of the Enzyme

**Figure 9:**
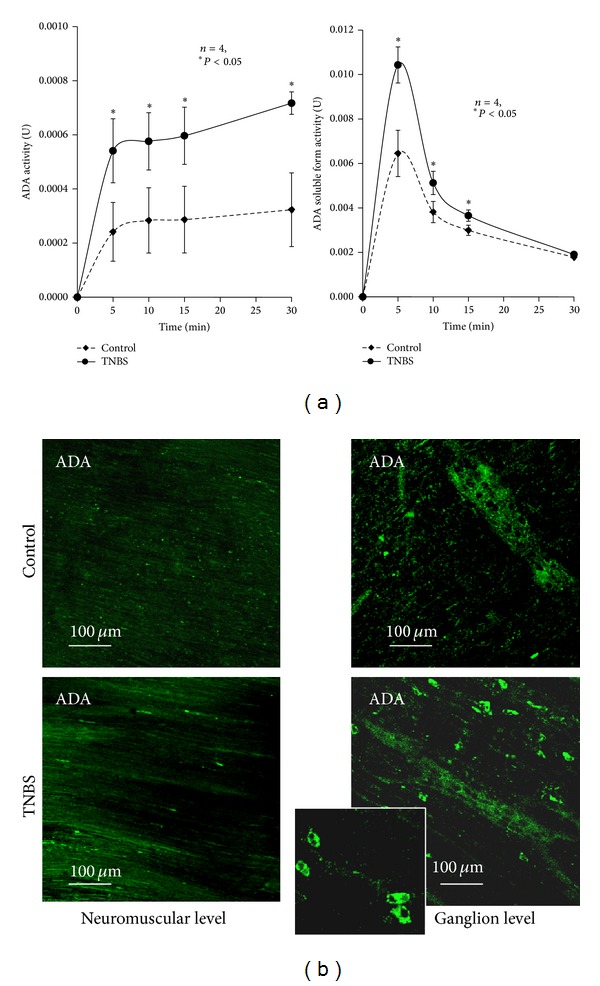
(a) Activity of ADA during the extracellular catabolism of adenosine in the LM-MP of the ileum of control and TNBS-injected rats. Adenosine (30 *μ*M) was incubated at zero time. Average results obtained in four experiments; the vertical bars represent the SEM and are shown when they exceed the symbols in size. *P < 0.05 (one-way ANOVA followed by Dunnett's modified* t*-test) represent significant differences as compared to control animals. (b) Localization of ADA immunoreactivity in single confocal images of whole-mount preparations of the longitudinal muscle-myenteric plexus of the ileum of control and TNBS-injected rats, taken both at ganglia and neuromuscular layers. Please note that ADA immunoreactivity is much more exuberant in preparations from TNBS-treated rats than in control animals. The figure insert details ADA staining in mononuclear inflammatory cells infiltrating myenteric ganglia.

The bath concentrations of adenosine (30 *μ*M) decrease progressively with time yielding to the formation of inosine and hypoxanthine in the LM-MP of the rat ileum (see [19]). The extracellular catabolism of ADO (30 *μ*M) was faster (*t*_1/2_ = 24 ± 3 min, *n* = 4) in TNBS-treated than in control preparations (*t*_1/2_ = 52 ± 10 min, *n* = 4). The rate of extracellular adenosine inactivation can be best appreciated by calculating adenosine deaminase (ADA) activity in the present experimental conditions, which is represented in Figure 9(a). Data indicate that the LM-MP of the ileum following an inflammatory insult has increasing amounts of ADA that we confirmed by immunofluorescence confocal microscopy (Figure 9(b)). ADA immunoreactivity was detected both at the ganglion level, as well as at the neuromuscular layer probably attached to smooth muscle fibers. In TNBS-treated preparations, ADA staining was also observed in mononuclear cells, most probably T lymphocytes and macrophages, infiltrating the myenteric plexus at the ganglion level [50–52]. Likewise, Antonioli and colleagues [53] demonstrated by Western blot analysis increases in the expression of ADA at the level of inflamed colonic tissues.

Like the ecto-5′-nucleotidase/CD73, extracellular ADA can be found attached to the plasma membrane (ecto-ADA) [20], as well as in soluble forms after proteolytic cleavage from cell membranes (exo-ADA) [21]. Both forms retain catalytic activity. Figure 9(a), also shows that the activity of the soluble form of ADA retained in the incubation fluid is highest during the first 5 min after removing the LM-MP preparation from the bath. Soluble ADA activity was not accompanied by any modification of lactate dehydrogenase activity, thus indicating that its activity is not due to damaged cells (see e.g., [19]). The soluble ADA activity was further increased in the incubation fluid which had been in contact for 45 min with preparations treated with TNBS. This implies that ADA is secreted in high amounts from the inflamed ileum, thus contributing to explaining the lack of adenosine neuromodulatory tonus. Even though inosine may accumulate in postinflammation ileitis as a result of increased extracellular adenosine deamination (see Figures 5 and 6), we failed to detect any variation on evoked [^3^H]-ACh release when inosine was applied to the incubation fluid of control and TNBS-treated preparations in concentrations as high as 1 mM (data not shown). Catabolic degradation of adenosine by soluble ADA may also result in the impairment of immune modulation by endogenous adenosine (via A_2A_ receptors on mononuclear cells, see Figure 2(b)) and the consequent worsening of inflammation and tissue injury [53].

## 4. Discussion and Conclusions

Enteric plasticity comprises a wide range of structural and/or functional changes in enteric neurons, glial cells, and pacemaker interstitial cells of Cajal, which are located between the muscle layers of gastrointestinal tract (GI). Different types of pathophysiological conditions drive to distinct adaptive responses. Enteric neurons are known to control all gastrointestinal functions. They are able to rapidly change their structure, function, or chemical coding to maintain the gut homeostasis (e.g., during inflammatory disorders) [52]. Despite heterogeneity of enteric adaptive responses, involvement of ubiquitous purine nucleotides, and nucleosides provides fine tuning control of peristaltic motor performance in the gut during stressful situations, such as sustained neuronal activity, ischemia, and inflammation (reviewed in [20]). Numerous studies have described the potential role of purinergic function regulators in inflammation and immunity [44, 54–56]. There is recent evidence that blockade of adenosine kinase, which results in increased endogenous levels of adenosine, downregulates the inflammatory response in experimental colitis [57]. In addition, Mabley et al. [58] showed that inosine resulting from the breakdown of adenosine effectively reduced the inflammatory response in a murine model of colitis. Despite this, we showed in this study that inosine accumulation might not be sufficient to explain the purinergic neuromodulatory changes observed in postinflammation ileitis. These, and many other findings, suggest that adenosine can mediate both proinflammatory (via A_1_ or A_2B_ receptors) and/or anti-inflammatory/immunosuppressant activities (mainly through A_2A_ and A_3_ receptors) in inflammatory gut diseases, and they have potential for the treatment of these diseases (reviewed in [44]). However, much is unknown to what extent the immune-modulatory role of purines during inflammation interferes with neuromodulation of the enteric nervous system [14].

Control of cellular activity by purines depends on the activation of two families of membrane-bound purinoceptors, P1 and P2, which are sensitive to adenosine and to adenine and/or uracil nucleotides, respectively. Interestingly, P1 and P2 purinoceptors are also present on immune cells and there is evidence that adenosine and ATP are generated at sites of inflammation (see, for a review [59]). Purinoceptors activation is fine-tuned regulated by the extracellular conversion of released adenine and uracil nucleotides leading to the formation of other biologically active products, namely, ADP/UDP and adenosine, via a cascade of membrane-bound ectonucleotidases [8, 60]. Thus, cellular expression and topology of ectonucleotidase enzymes, as well as factors that extrinsically affects their catalytic activity (e.g., ionic concentration, nucleotide binding) create gradients of adenine nucleotides and their breakdown products, which may discriminate the nearby purinoceptor that is more likely to be activated. For instance, under basal conditions ATP transiently facilitates acetylcholine from nonstimulated myenteric neurons via prejuctional P2X (probably P2X2) receptors; however, increases in the amount of ATP released from stimulated neurons create conditions favourable to ADP accumulation at the myenteric synapse, leading to downregulation of transmitter release via the P2Y_1_ receptor [12]. Strategic manipulation of the activity of ectonucleotidases has been proposed as a novel therapeutic approach to modify pathogenic purinergic signalling cascades (reviewed in [61]).

At the myenteric plexus, the rate limiting enzyme responsible for extracellular adenosine formation from released adenine nucleotides is ecto-5′-nucleotidase/CD73 [12, 18]. This enzyme acts in a concerted manner with adenosine inactivation pathways, both cellular uptake and adenosine deaminase (ADA), to control extracellular levels of the nucleoside and, thereby, the activation of co-localized P1 receptors [19]. Interestingly, although much less has been explored in functional terms, the enzymes most relevant to control adenosine levels in the extracellular milieu, both ecto-5′-nucleotidase/CD73 and ADA, may be cleaved from their anchor to the plasma membrane while retaining their catalytic activity in the soluble form [20]. Coincidently or not, significant increases in ecto-5′-nucleotidase/CD73 and ADA mRNA expression were observed in inflamed colonic tissues [62]. Fragile docking of these enzymes to the plasma membrane is more likely to occur within the context of inflammatory reactions, given that recruitment of inflammatory cells release a huge number of cytokines, chemokines and enzymes, some of these with proteolytic activity. One of these enzymes is glycosylphosphatidylinositol-specific phospholipase, which plays a role in inflammation since it can hydrolyse the GPI anchor of several membrane proteins and its hydrolytic products up-regulate cytokine expression in macrophages [63]. The widespread distribution of soluble forms of ecto-5′-nucleotidase/CD73 and ADA create conditions to unbalanced bulk production and/or inactivation of adenosine way from its original location, thus affecting mass organ function. Likewise, inflammatory infiltrates including T lymphocytes, which are endowed with NTPDase1/CD39 and ecto-5′-nucleotidase/CD73 enzymes, may additionally contribute to adenosine accumulation disturbance and to unpredictable P1-receptor-mediated responses in inflamed tissues. In light of these compelling hypotheses, this work was designed to investigate the kinetics of the catabolism of adenine nucleotides and adenosine accumulation in the myenteric plexus of the ileum following an inflammatory insult.

Data from this study evidenced a disparity between higher amounts of ATP detected in the extracellular fluid released from TNBS-treated ileal preparations and deficits in the adenosine levels measured in the same samples, both under baseline conditions and after electrical stimulation. This puzzling contradiction has not been detected before. On the contrary, indirect evidences made by other authors led to the hypothesis that the disruption of ATP release could be due to mitochondrial dysfunction induced by inflammatory mediators [40, 45], which was not confirmed in the present study. In postinflammation ileitis, higher ATP amounts can be originated from infiltrating immune and non-neuronal cells, such as glial cells, interstitial cells of Cajal and smooth muscle contractions (see e.g., [7]; reviewed in [64]), considering the proposed inflammatory neuronal dysfunction. Hemichannels containing pannexins may act as conduits for ATP release from non-neuronal cells in response to physiological and pathological stimuli. These channels are able to form signalling complexes with the purinergic P2X7 receptor, which activation leads to large pore formation and consequently to further release of ATP [65, 66]. These authors proposed a model where high extracellular levels of ATP chronically activate neuronal P2X7, pannexins, and caspases. This relationship between P2X7 and pannexins has been related to the loss of enteric neurons during inflammatory conditions [52, 66, 67]. Moreover, our data add some information to explain the impairment of purinergic signalling during intestinal inflammation detected in other reports [14, 39, 40] which has been expanded in this study by showing a lack of adenosine neuromodulatory control of acetylcholine release from postinflammation myenteric plexus. We provided compelling evidences that most of the adenosine originated in the inflamed myenteric plexus of the rat ileum was derived from activated ICC's by the release of the nucleoside as such via dipyridamole-sensitive equilibrative transporters. This also agrees with previous findings from our group showing that adenosine formation from released ATP, via the ecto-nucleotidase cascade, contributes only partially to the total interstitial adenosine concentration in the myenteric plexus of healthy rats [19].

The apparent disturbance of adenosine generated from the extracellular catabolism of released ATP may be due to feed-forward inhibition of ecto-5′-nucleotidase/CD73 as a consequence of high extracellular levels of ATP, and particularly ADP, accumulated in the inflamed myenteric synapse (cf. [47]). ATP and ADP are competitive inhibitors of ecto-5′-nucleotidase/CD73 with inhibition constants in the low micromolar range. This indicates that they can bind to the active site of this enzyme, like AMP, but they cannot be hydrolysed [11, 68]; recovery of ecto-5′-nucleotidase/CD73 activity occurs once extracellular ATP and ADP levels decrease below the micromolar range. Redistribution of NTPDase2 from ganglion cell bodies, which is the preferential localization of this enzyme in healthy animals, towards myenteric nerve terminals affords the most probable explanation for the unpredicted ADP accumulation in postinflammation neuromuscular synapse. This is possible because NTPDase2 is a preferential nucleoside triphosphatase hydrolysing ADP 10 to 15 times less efficiently than ATP [8], and we detected no changes in the distribution of NTPDase3 and NTPDase8 present in the myenteric plexus. Notably, no evidence of NTPDase1 expression was detected in the rat myenteric plexus, besides a few blood vessels. Thus, during postinflammation ileitis the hydrolysis of ATP into adenosine at the myenteric synapse may be transiently interrupted by feed-forward inhibition of ecto-5′-nucleotidase/CD73, which makes a shift from P1 receptors activation (by adenosine) to a preferential P2 receptors activation (by ATP or ADP). These data are in agreement with the hydrolysis of ATP by NTPDase2 and ecto-5′-nucleotidase/CD73 in the rat liver [69]. Indeed, depending with which NTPDase subtype ecto-5′-nucleotidase/CD73 co-localizes different amounts of adenosine will be generated [69]. The functional interpretation of these findings in order to justify the increase in gastrointestinal motility may be complicated by disturbances in compartmentalization originated by the release of significant amounts of soluble forms of the enzyme [11, 20]. Nevertheless, our results fully agree with other authors suggesting that ecto-5′-nucleotidase/CD73 might play a significant role in the modulation of purinergic signalling during enteric inflammation [44, 55].

Previous findings from our group demonstrated that extracellular deamination by ADA is the most efficient mechanism regulating synaptic adenosine levels and, consequently, tonic activation of facilitatory A_2A_ receptors in myenteric nerve terminals of healthy rats [18]. Besides the very high level of activity of ecto-ADA, a less-efficient nucleoside transport system sensitive to dipyridamole may also contribute to inactivation of extracellular adenosine. Owing to the effectiveness of both inactivation mechanisms, endogenous adenosine actions may be restricted to the release/production region at the myenteric cholinergic synapse, while exogenously added adenosine seems to activate preferentially extrajunctional inhibitory A_1_ receptors [16, 18]. A question remains unanswered regarding the tonic effect of endogenous adenosine on low affinity excitatory A_3_ receptors, although selective agonists to this receptor have been shown to beneficially influence inflammation in experimental models [53, 70]. Tandem localization of adenosine A_3_ (on cell bodies) and A_2A_ (on nerve varicosities) receptors along myenteric neurons explains why the A_3_ receptor activation may be prevented by ZM 241385 (50 nM), a selective A_2A_ receptor antagonist with low affinity for A_3_ receptors (*K*_*i*_ > 10 *μ*M). This implies that endogenous adenosine acts preferentially on prejunctional A_2A_ under normal conditions making the activation of low affinity A_3_ receptors by adenosine high unlikely during the postinflammatory phase of TNBS-ileitis due to suppression/inactivation of the nucleoside (this study). The putative activation of A_3_ receptors by endogenously formed inosine via ADA also cannot explain the loss of the neuromodulatory influence of nucleosides in postinflammation ileitis. While we are uncertain regarding the extracellular levels of adenosine at both particular locations, both* in vivo* and* in vitro* models suggest that the balance between inhibitory A_1_ and excitatory A_2A_ may be important in regulating intestinal motility. For instance, de Man et al. reported that chronic intestinal inflammation enhanced enteric contractile activity in part due to a loss of the cholinergic neuromodulation mediated by A_1_ receptors [14]. It is worth noting that human post-ganglionic myenteric neurons co-express adenosine A_1_ and A_2A_ receptors, which exhibit a heterogenous distribution [71]. Therefore, there is a considerable interest in the neuroprotective effects exerted by adenosine during inflammatory (and ischemic) insults, and it is conceivable that adenosine accumulation disturbances may contribute to enteric excitability during pathological conditions.

Besides the proposed interruption of the ectonucleotidase cascade at the level of ecto-5′-nucleotidase/CD73 leading to AMP accumulation and low adenosine formation in the inflamed myenteric plexus, our data also show that ADA activity is significantly enhanced in postinflammation ileitis thus contributing to decrease the extracellular adenosine levels and, thereby, tonic activation of P1 receptors. ADA is the enzyme responsible for the deamination of adenosine into inosine. This enzyme has a wide phylogenetic distribution and its amino acid sequence is highly conserved, suggesting that ADA is a key enzyme in purine metabolism. Besides the classical intracellular form, ADA is reported to bind to the cell surface of T lymphocytes, via the activation cell marker CD26 [72]. The presence of ADA in nerve terminals has also been demonstrated in many brain regions and it was hypothesize that ADA-containing terminals may release adenosine or the enzyme itself, which could serve to regulate purinergic neurotransmission [73, 74]. In the rat myenteric plexus, we first described that ADA activity increased in bath samples collected after tissue stimulation in parallel with the interstitial accumulation of adenosine and its metabolites [19]. This implies that the amount of adenosine detected following stimulation of the preparation was largely underestimated as compared to the levels of the nucleoside in the biophase. Secretion of ADA may occur in several diseases, namely those affecting hematopoietic and immune [75]. To our knowledge, this is the first study reporting an increase in the activity of soluble ADA in the chronic inflamed myenteric plexus of the rat ileum. Our findings unravel the mechanism by which inhibition of ADA might attenuate inflammation in experimental colitis [76].

In conclusion, data indicate that postinflammation ileitis suppresses adenosine neuromodulation, which may contribute to increase gastrointestinal motility. Impairment of adenosine neuromodulation is most probably due to deficient accumulation of the nucleoside at the myenteric synapse despite the paradoxical increase in ATP release. Discrepancy between ATP outflow and adenosine deficiency in the chronic inflamed ileum can be ascribed to feed-forward inhibition of ecto-5′-nucleotidase/CD73 by high extracellular levels of ATP and/or ADP. Interestingly, redistribution of NTPDase2, but not of NTPDase3, from ganglion cell bodies to myenteric nerve terminals leads to preferential ADP accumulation from released ATP, thus contributing to prolong inhibition of muscle-bound ecto-5′-nucleotidase/CD73 and to delay adenosine formation at the inflamed neuromuscular synapse. On the other hand, depression of endogenous adenosine accumulation may also occur due to enhancement of ADA activity. We observed a remarkably increase in the activity of soluble forms of ecto-5′-nucleotidase/CD73 and ADA in the postinflammatory phase of TNBS-ileitis, which may contribute to unbalanced bulk production and/or inactivation of adenosine way from its location in the native tissue thus affecting organ function. Thus, our findings suggest that pharmacological inhibition of ADA, and/or the use of AMP-derived prodrugs able to selectively activate P1 receptor co-localized with ecto-5′-nucleotidase/CD73, represent promising therapeutic strategies to ameliorate motility disturbances and immune reactivity inherent to inflammatory enteric disorders.

## Figures and Tables

**Table 1:** Primary and secondary antibodies used in immunohistochemistry experiments.

Antigen	Code	Species	Dilution	Supplier
Primary antibodies				
Adenosine receptor A_1_	AB1587P	Rabbit	1 : 50	Chemicon
Adenosine receptor A_2A_	A2aR21-A	Rabbit	1 : 150	Alpha Diagnostics
NTPDase1	rN1-6_L_I_4_	Rabbit	1 : 1000	http://ectonucleotidases-ab.com/
NTPDase2	rN2-6_L_	Rabbit	1 : 400	http://ectonucleotidases-ab.com/
NTPDase3	rN3-1_L_I_5_	Rabbit	1 : 150	http://ectonucleotidases-ab.com/
NTPDase8	rN8-8_C_I_5_	Guinea-pig	1 : 600	http://ectonucleotidases-ab.com/
Ecto-5′-nucleotidase	rNu-9_L_I_5_	Rabbit	1 : 1000	http://ectonucleotidases-ab.com/
ADA	sc-25747	Rabbit	1 : 50	Santa Cruz
Secondary antibodies				
Alexa Fluor 488 anti-rb	A-21206	Donkey	1 : 1000	Molecular probes
TRITC 568 anti-gp	706-025-148	Donkey	1 : 150	Molecular probes

## Abbreviations

ACh: Acetylcholine
ADA: Adenosine deaminase
ADO: Adenosine
ADP: Adenosine diphosphate
AMP: Adenosine monophosphate
ATP: Adenosine triphosphate
CNS: Central nervous system
DSS: Dextran sodium sulphate
E-NTPDase: Ectonucleoside triphosphate diphosphohydrolase
E-NPP: Ecto-nucleotide pyrophosphatases/phosphodiesterases
EFS: Electrical field stimulation
ENS: Enteric nervous system
GI: Gastrointestinal
GPI: Glycosylphosphatidylinositol
HPLC: High performance liquid chromatography
HX: Hypoxanthine
IBD: Inflammatory bowel disease
ICC's: Interstitial cells of Cajal
INO: Inosine
LM-MP: Longitudinal muscle-myenteric plexus
Min: Minutes
PBS: Phosphate saline buffer
PLP: Periodate-lysine-paraformaldehyde
TTX: Tetrodotoxin
TNBS: 2,4,6-trinitrobenzenesulfonic acid.
